# Rapid progression to toxic epidermal necrolysis following switch of PD-1 inhibitors: a case report

**DOI:** 10.3389/fmed.2026.1789285

**Published:** 2026-05-18

**Authors:** Meiling Huang, Yunfeng Guan, Zhenyi Yang

**Affiliations:** 1 Department of Radiotherapy, Huzhou Central Hospital, Affiliated Central Hospital Huzhou University, Huzhou, Zhejiang, China; 2 Department of Radiotherapy, Huzhou Central Hospital, Fifth Affiliated Clinical Medical College of Zhejiang Chinese Medical University, Hangzhou, China

**Keywords:** immune-related adverse events, pembrolizumab, Stevens-Johnson syndrome, tislelizumab, toxic epidermal necrolysis

## Abstract

Immune checkpoint inhibitors (ICIs) have been widely used in clinical practice in recent years. Stevens-Johnson syndrome (SJS) and toxic epidermal necrolysis (TEN) are rare, potentially life-threatening cutaneous adverse reactions associated with ICIs. This report describes a 69-years-old male with recurrent hypopharyngeal cancer who experienced only recurrent Grade 1 maculopapular rash during treatment with tislelizumab combined with chemotherapy and subsequent maintenance therapy. However, within 2 days after switching to pembrolizumab, his condition deteriorated rapidly into TEN, involving over 95% of the body surface area and multiple mucosal sites including the oral cavity and conjunctivae. Skin biopsy revealed epidermal necrosis, subepidermal clefts, and infiltration of CD4 and CD8 positive T lymphocytes. Immunohistochemistry demonstrated positive PD-L1 expression but negative PD-1 expression. These pathological findings suggested that alterations in the local cutaneous immune microenvironment following sequential administration of different PD-1 inhibitors might play a critical role in the fulminant progression of TEN.

## Introduction

Programmed cell death-1 (PD-1) inhibitors have revolutionized the treatment of multiple malignancies and achieved durable clinical responses in a considerable proportion of patients. Nonetheless, the widespread immune activation triggered by PD-1 blockade can lead to a variety of immune-related adverse events (irAEs). Cutaneous irAEs represent one of the most common irAEs, affecting 30%–50% of patients receiving PD-1 inhibitors (1, 2). While most cutaneous reactions are mild and manageable, severe cutaneous adverse reactions (SCARs), especially Stevens-Johnson syndrome (SJS) and toxic epidermal necrolysis (TEN), are rare but life-threatening complications characterized by extensive epidermal necrosis, mucosal involvement, and high mortality.

In recent years, increasing cases of immunotherapy-related SJS/TEN have been documented. A recent systematic review summarized 47 published articles involving 50 cases of immunotherapy-induced SJS/TEN, of which 41 cases were associated with PD-1 inhibitors, including pembrolizumab (20 cases), nivolumab (12 cases), sintilimab (8 cases), and toripalimab (1 case) (3). However, the underlying immunological mechanisms of PD-1 inhibitor-related SJS/TEN remain incompletely understood, and are thought to involve dysregulated immune activation, keratinocyte damage, and abnormal T-cell-mediated cytotoxicity.

Notably, switching between different PD-1 inhibitors is not uncommon in clinical practice, often performed due to insufficient efficacy, intolerable mild irAEs, or drug availability. However, the safety profile and potential risks of PD-1 inhibitor switching, especially in patients with pre-existing mild cutaneous irAEs, remain poorly defined. To date, no case has been reported in which switching PD-1 inhibitors led to abrupt exacerbation of pre-existing mild cutaneous reactions and rapid progression to fatal TEN.

Here, we report a patient with hypopharyngeal carcinoma who developed recurrent grade 1 maculopapular rash during treatment with tislelizumab, but experienced rapid progression to definitive TEN after the first cycle of switching to pembrolizumab. This case highlights the clinical complexity and potential risks of PD-1 inhibitor switching in patients with pre-existing mild cutaneous irAEs, and underscores the need for heightened vigilance in clinical management.

## Clinical data

A 69-years-old male with an ECOG performance status of 1, no chronic underlying diseases, and no history of drug allergies was diagnosed with hypopharyngeal squamous cell carcinoma in May 2015 and underwent radical surgery. Postoperative pathology confirmed moderately differentiated squamous cell carcinoma, pathological stage pT3N1M0. He received postoperative adjuvant radiotherapy and regular follow-up.

**FIGURE 1:**
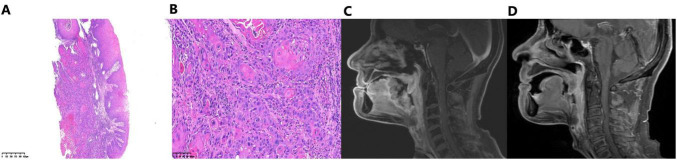
Diagnostic pathology and radiological response to chemotherapy combined with PD-1 inhibitor. **(A,B)** Histopathological analysis of the recurrent hypopharyngeal mass (hematoxylin and eosin staining). The morphology is consistent with the diagnosis of well-differentiated squamous cell carcinoma. **(C)** Baseline Neck MRI demonstrating an irregular, heterogeneously enhancing soft tissue mass extending from the tongue base to the larynx. **(D)** Follow-up MRI scan post-treatment indicating partial response.

In March 2025, the patient presented with a 1-week history of sore throat. Laryngoscopy revealed a pharyngeal neoformation, and biopsy confirmed well-differentiated squamous cell carcinoma (Figures 1A, B). Neck MRI showed an irregular soft-tissue lesion with heterogeneous enhancement extending from the tongue base to the larynx (Figure 1C). Chest and abdominal imaging showed no distant metastasis. The final diagnosis was recurrent hypopharyngeal carcinoma (rT4N0M0).

After multidisciplinary team (MDT) discussion, first-line combined chemoimmunotherapy was initiated in March 2025: cisplatin 35 mg intravenously (days 1–3) + albumin-bound paclitaxel 350 mg intravenously (day 1) + tislelizumab 200 mg intravenously (day 1), every 3 weeks for 6 cycles. Five days after the sixth cycle, the patient developed CTCAE 5.0 Grade 1 pruritic scattered maculopapular rash on the trunk, which resolved with antihistamines. Other adverse events included Grade 2 leukopenia and Grade 1 nausea/vomiting. Post-treatment efficacy evaluation showed partial response (PR) (Figure 1D).

In July 2025, the patient received maintenance tislelizumab 200 mg every 3 weeks for 2 cycles. Recurrent Grade 1 maculopapular rash occurred on the trunk after each cycle and improved with symptomatic treatment. In September 2025, the PD-1 inhibitor was switched to pembrolizumab 200 mg with no wash-out period due to recurrent mild cutaneous adverse events.

**FIGURE 2:**
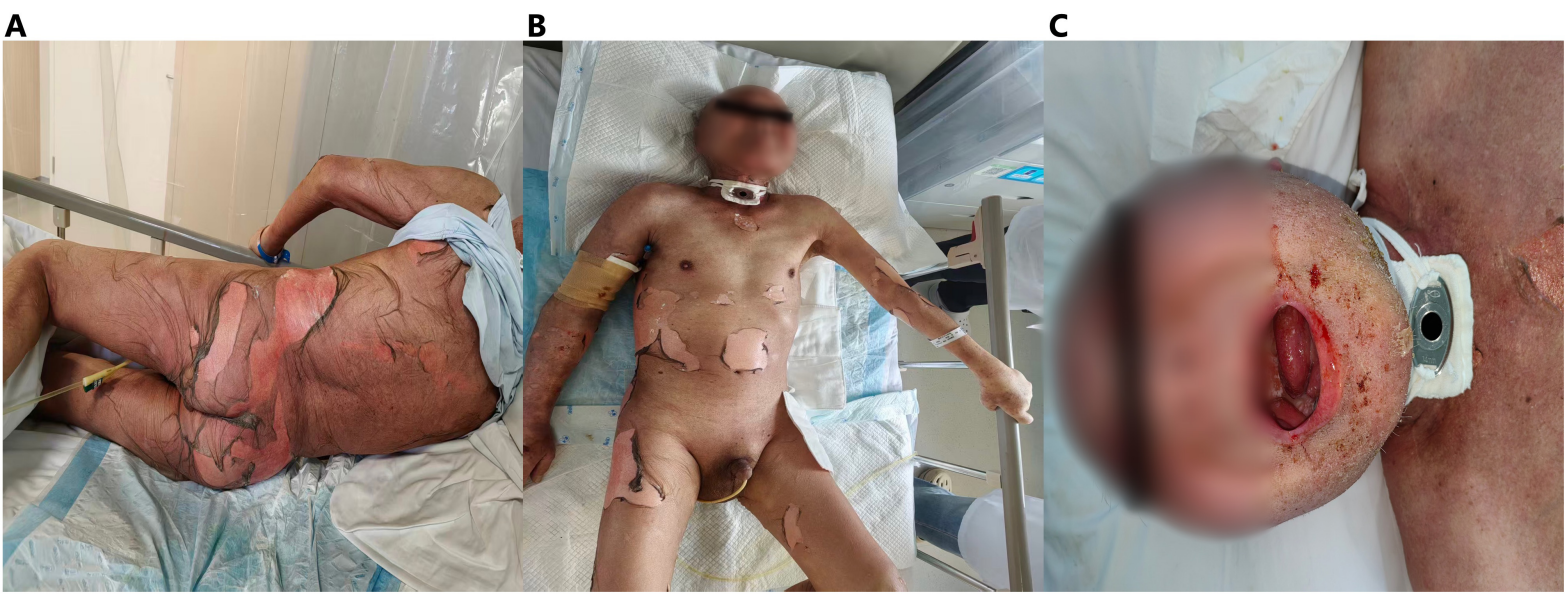
Skin and oral mucosa injury. **(A,B)** Widespread flaccid blisters of varying sizes and extensive epidermal detachment with erosions are seen on the face, trunk, and extremities. **(C)** The oral mucosa had erosions.

Within 48 h of pembrolizumab administration, the patient rapidly developed extensive dark-red confluent macules on the face, extremities, and trunk, with flaccid blisters and epidermal detachment, progressing to widespread erosion. Nikolsky’s sign was positive. Mucosal involvement included conjunctival erosion, oral mucosal ulceration, and genital mucosal erosion (Figures 2A–C). Epidermal detachment involved >95% of total body surface area (BSA), assessed by the rule of nines.

**FIGURE 3:**
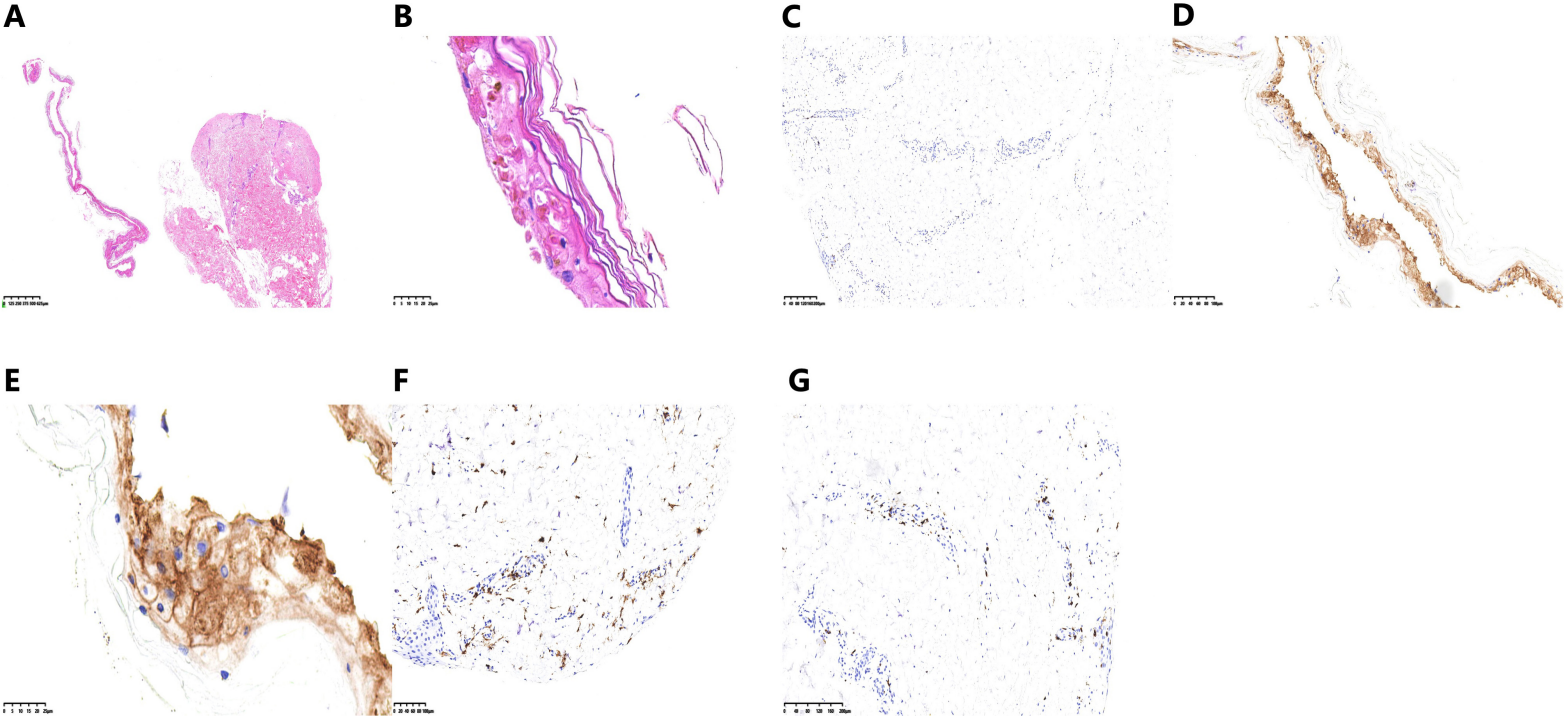
The biopsy of the skin lesion of left lower limb. **(A,B)** Hematoxylin and eosin staining shows epidermal-dermal separation and epidermal necrosis. **(C)** PD-1 immunohistochemistry reveals negative expression in the basal layer of the epidermis. **(D,E)** PD-L1 immunohistochemistry demonstrates positive expression in the basal layer of the epidermis. **(F)** CD4 immunohistochemistry shows infiltration of CD4+ T lymphocytes. **(G)** CD8 immunohistochemistry shows infiltration of CD8+ T lymphocytes.

Skin biopsy from the left lower limb showed full-thickness epidermal necrosis and subepidermal clefts, diagnostic of TEN (Figures 3A, B). Immunohistochemical staining was performed on neutral formalin-fixed, paraffin-embedded skin tissue using ready-to-use reagents on the Leica Bond 3 platform. Antibody details: CD4 (clone SP35, ready-to-use), CD8 (clone OTIR3D5, ready-to-use), PD-1 (clone MX033, ready-to-use), PD-L1 (clone E1L3N, ready-to-use). Interpretation criteria: membrane staining in immune cells was considered positive. Results showed negative PD-1 expression in epidermal and infiltrating immune cells (Figure 3C); moderate-to-strong PD-L1 expression in epidermal keratinocytes and perilesional immune cells (Figures 3D, E); prominent infiltration of CD4+ helper T cells (Figure 3F) and CD8+ cytotoxic T cells (Figure 3G) in the dermal-epidermal junction and dermis, consistent with immune-mediated epidermal cytotoxicity in PD-1 inhibitor-related TEN.

Following urgent MDT consultation (Dermatology, Burn Surgery, Critical Care Medicine, Clinical Nutrition), immediate rescue treatment was initiated: intravenous methylprednisolone 80 mg once daily, intravenous immunoglobulin (IVIG) 0.4 g/kg once daily for 5 consecutive days, and subcutaneous adalimumab 80 mg as a single dose. The decision was based on the fulminant TEN severity, hospital drug availability, and the patient’s critical clinical status. The patient was admitted to a laminar airflow isolation room, with standardized supportive care including fluid resuscitation, electrolyte balance maintenance, parenteral nutrition, sterile skin/mucosal care, and infection prophylaxis.

During treatment, the patient developed secondary bacterial and fungal bloodstream infections; targeted anti-infective therapy was administered based on blood culture and drug-sensitivity results. Despite aggressive intensive care, epidermal detachment and systemic infection progressed. The patient died of sepsis 2 weeks after TEN onset.

## Discussion

Chemotherapy plus PD-1 inhibitors is a first-line standard regimen for recurrent/metastatic head and neck squamous cell carcinoma (4). After multidisciplinary team discussion, this patient with recurrent hypopharyngeal cancer received tislelizumab combined with albumin-bound paclitaxel and cisplatin, and achieved a partial response after 6 cycles of treatment.

Anti-PD-1 therapy is frequently associated with inflammatory skin reactions, ranging from mild maculopapular rashes to severe Stevens-Johnson syndrome/toxic epidermal necrolysis (SJS/TEN)-like eruptions (3). SJS/TEN are well-recognized drug-induced type IV hypersensitivity reactions (5). Compared with non-immune checkpoint inhibitor (ICI)-related cases, ICI-associated SJS/TEN typically involves a smaller body surface area but carries a significantly higher risk of severe outcomes (6, 7).

After 6 cycles of combination therapy, the patient developed grade 1 maculopapular rash during tislelizumab treatment, but rapidly progressed to TEN within 48 h after switching to pembrolizumab. Assessment using the Naranjo Adverse Drug Reaction Probability Scale yielded a total score of 9 for PD-1 inhibitors, indicating a probable causal relationship between the cutaneous adverse reaction and PD-1 inhibitor administration (Supplementary Table 1). A systematic review by Zhou et al. confirmed that ICI-associated SJS/TEN has a characteristic latency period, and some patients present with low-grade rashes as a prodromal sign (3). Notably, the pattern observed in this case–progression from a mild reaction to the first PD-1 inhibitor to fulminant TEN immediately after switching–is extremely rare in clinical practice.

The pathogenesis of traditional drug-induced SJS/TEN is well-defined as a T-cell-mediated type IV delayed hypersensitivity reaction that induces massive keratinocyte apoptosis (8–10). Established facts also include that the PD-1/PD-L1 pathway is critical for maintaining peripheral immune tolerance, and PD-L1 expression in normal skin is low but can be upregulated under inflammation as a compensatory inhibitory mechanism (11–16). Anti-PD-1 therapy removes immune suppression and may trigger excessive immune activation in skin tissue (3, 17, 18). The PD-L1 positivity in our patient’s skin biopsy is consistent with this compensatory response.

Given the absence of a wash-out period between tislelizumab and pembrolizumab, the clinical course supports an immunological continuum or priming effect rather than a *de novo* reaction to pembrolizumab alone. We hypothesize a synergistic toxic effect between the two PD-1 inhibitors, which remains mechanistically speculative: Tislelizumab may have primed the immune system into a hyperactivated state, as indicated by recurrent grade 1 rash. Subsequent pembrolizumab administration may have synergistically enhanced T-cell activation, leading to explosive disease progression. The negative PD-1 staining suggests full receptor occupancy or downregulation by PD-1 inhibitors, abolishing the compensatory inhibitory signal of PD-L1 and causing immune homeostasis collapse. Uncontrolled cytotoxic T-cell infiltration then drives massive keratinocyte death and rapid TEN progression. All above mechanistic interpretations are hypothetical and require validation by further large-sample studies.

Due to the rarity of PD-1 inhibitor-induced SJS/TEN, its management follows established protocols for traditional drug-induced TEN, focusing on supportive care including isolation, fluid and protein replacement, and close monitoring. First-line systemic therapies include glucocorticoids, intravenous immunoglobulin, cyclosporine, and TNF-α antagonists (19–23); refractory cases may require plasma exchange or combination regimens (24). Despite aggressive multidisciplinary treatment, the patient died of progressive skin lesions and secondary sepsis. The fatal outcome was multifactorial: immune homeostasis collapse induced by PD-1 inhibitor switching, and chemotherapy-related myelosuppression (persistent severe neutropenia) that increased infection susceptibility.

This case carries important clinical implications. Switching PD-1 inhibitors merely for mild cutaneous adverse events is not supported by current evidence. Different PD-1 inhibitors may share cross-reactive risks of severe cutaneous toxicity, so intra-class switching cannot be regarded as a safe strategy. For patients with pre-existing mild skin irAEs related to PD-1 inhibitors, clinicians should strengthen monitoring, avoid abrupt switching without a wash-out period, and make clinical decisions with extreme caution. This case also highlights the need for early recognition of prodromal rashes and prompt intervention to prevent fulminant progression to life-threatening SJS/TEN.

This study has several limitations inherent to a single case report. First, the mechanistic hypothesis of synergistic toxicity and immune continuum is speculative and cannot be verified by experimental data. Second, the lack of serial immunological monitoring (e.g., dynamic T-cell subsets, cytokine levels) limits in-depth analysis of the immune changes during PD-1 inhibitor switching. Third, the single-case design restricts the generalizability of the findings, and the causal relationship between PD-1 inhibitor switching and rapid TEN progression cannot be definitively confirmed. Further research with larger cohorts and basic experimental studies are needed to validate these observations and clarify the underlying mechanisms.

## Data Availability

The original contributions presented in this study are included in the article/Supplementary material, further inquiries can be directed to the corresponding author.

## Supplementary material

Supplementary Table 1 Naranjo adverse drug reaction probability scale.
