# Supplementary material for: Rapid progression to toxic epidermal necrolysis following switch of PD-1 inhibitors: a case report
Source: Front Med (Lausanne). 2026 May 18;13:1789285. doi: 10.3389/fmed.2026.1789285 (PMC13222964; doi:10.3389/fmed.2026.1789285)
Supplement: Supplementary Table 1 — Naranjo adverse drug reaction probability scale. [file Data_Sheet_1.pdf]

Naranjo Adverse Drug Reaction Probability Scale				
Question	Yes	No	Do Not Know	Score
1.Are there previous conclusive reports on this reaction?	+1	0	0	+1
2.Did the adverse event appear after the suspected drug was administered?	+2	-1	0	+2
3.Did the adverse reaction improve when the drug was discontinued or a specific antagonist was administered?	+1	0	0	0
4.Did the adverse event reappear when the drug was re-administered?	+2	-1	0	+2
5.Are there alternative causes (other than the drug) that could on their own have caused the reaction?	-1	+2	0	+2
6.Did the reaction reappear when a placebo was given?	-1	+1	0	0
7.Was the drug detected in blood (or other fluids) in concentrations known to be toxic?	+1	0	0	0
8.Was the reaction more severe when the dose was increased or less severe when the dose was decreased?	+1	0	0	0
9.Did the patient have a similar reaction to the same or similar drugs in any previous exposure?	+1	0	0	+1
10.Was the adverse event confirmed by any objective evidence?	+1	0	0	+1
TOTAL SCORE	9			
